# Most vulnerable diet and health profiles: identifying countries at risk through a three-theme clustering

**DOI:** 10.3389/fpubh.2025.1569755

**Published:** 2025-07-07

**Authors:** Ahmet Murat Günal, Salim Yılmaz, Sedat Arslan

**Affiliations:** ^1^Department of Nutrition and Dietetics, Faculty of Health Sciences, Haliç University, İstanbul, Türkiye; ^2^Department of Healthcare Management, Faculty of Health Sciences, Acibadem Mehmet Ali Aydinlar University, İstanbul, Türkiye; ^3^Department of Nutrition and Dietetics, Faculty of Health Sciences, Bandirma Onyedi Eylul University, Balikesir, Türkiye

**Keywords:** food insecurity, dietary habits, healthcare services, life expectancy, health outcomes, clustering analysis

## Abstract

**Background:**

Global disparities in dietary habits, healthcare services, and life expectancy continue to affect vulnerable populations, particularly in low-and middle-income countries. Malnutrition, inadequate healthcare infrastructure, and diet-related chronic diseases contribute significantly to these disparities, necessitating targeted public health interventions.

**Objectives:**

This study aims to identify the most vulnerable countries in terms of diet, health, and life expectancy using a three-theme clustering approach. The study categorizes countries based on nutrition and diet, health and disease burden, and healthcare access and life expectancy to determine those at the highest risk.

**Methods:**

A cross-sectional analysis was conducted using 2019 data from 168 countries. A k-means clustering algorithm was applied to classify countries into three risk-based clusters for each theme. The Jaccard similarity coefficient was used to evaluate cluster overlap, and statistical significance was assessed through robust regression models and the Kruskal–Wallis H test. Data processing and visualization were performed using RStudio.

**Results:**

The analysis identified Cluster 3 as the most vulnerable group, comprising 45 countries with high similarity across all three themes. These countries, predominantly in Africa and Asia, exhibited low daily animal protein intake (<20 g/day), high rates of diet-related diseases, and limited access to healthcare. The healthcare coverage index in these countries ranged from 45.33 to 84.48, with life expectancy as low as 63.39 years. The findings highlight critical inequalities in global health, emphasizing the need for targeted interventions.

**Conclusion:**

This study underscores the urgent need for improved nutrition policies and healthcare investments in high-risk regions. Addressing malnutrition, enhancing healthcare services, and implementing targeted public health initiatives are crucial for reducing global health disparities and improving outcomes in vulnerable populations.

## 1 Introduction

Understanding the global impact of malnutrition and diet-related diseases is essential for developing effective public health strategies and implementing successful interventions that strengthen community health (1). Despite the United Nations’ goal to eliminate hunger by 2030, approximately 820 million individuals continue to experience hunger or malnutrition-related health problems each year (2, 3). Dietary patterns and health profiles directly influence individuals’ quality of life and longevity, forming the foundation of societal well-being (4). However, in countries with inadequate health systems, these fundamental elements are compromised by malnutrition, underscoring the urgent need for comprehensive nutrition policies.

Globally, malnutrition continues to affect vulnerable populations. Among the 264 million women with anemia, 22.3% of their children experience stunted growth, and 6.8% are underweight (5). The world has yet to overcome this challenge. For example, India has the highest number of undernourished individuals globally, accounting for 15% of its total population (6). The 2023 Global Hunger Index revealed that India ranked 107th out of 121 countries (7). Furthermore, the 2020 Global Nutrition Report indicated that 37.9% of children aged <5 years were stunted, and 20.8% were classified as underweight (3). These statistics highlight the severe consequences of nutritional deficiencies not only at the individual level but also for society at large, highlighting the need for improved health policies in these countries.

Low animal protein intake can cause reduced muscle mass, delayed wound healing, weakened immune function, and impaired overall body functions (8). Moreover, inadequate meat consumption may lead to deficiencies in essential nutrients, including vitamin B12, iron, zinc, and selenium (9). Furthermore, low carbohydrate consumption can impair kidney function and reduce bone density (10). Deficient fat intake can result in a weakened immune system, skin and hair health deterioration, hormonal imbalances, and disrupted neurological functions (11). Insufficient fruit consumption limits access to fiber, vitamins, and antioxidants (12). Dairy products are rich sources of calcium and protein (13), whereas vegetables are abundant in fiber, vitamins, minerals, and phytochemicals (14). These nutritional deficiencies adversely affect not only individual health but also the broader national health profile, underscoring the critical significance of focusing on comprehensive national nutrition policies (15).

A body mass index (BMI) outside the ideal range is a reflection of imbalanced or inadequate nutrition and is linked to serious health consequences. While a BMI exceeding the upper threshold is commonly associated with conditions such as obesity, diabetes, cardiovascular diseases, and specific types of cancer (16), a BMI below the lower accepted limit is equally detrimental to health. Underweight individuals, particularly those with severe malnutrition, are at increased risk of immune system suppression, osteoporosis, anemia, and sarcopenia, which can lead to frailty and heightened susceptibility to infections (17, 18). Chronic energy or nutrient deficiencies, often seen in undernourished populations, contributes to menstrual irregularities and reduced fertility (19). Furthermore, prolonged undernutrition is linked to cachexia, a condition characterized by severe muscle wasting, commonly observed in individuals with advanced chronic diseases such as cancer or tuberculosis (20). These highlight the bidirectional risks of BMI deviations and emphasize the necessity of balanced nutrition to prevent both overnutrition and undernutrition-related health disorders.

Excessive alcohol consumption triggers various health issues, from liver disease to alcoholism, and even specific cancers (21). Mortality due to protein–energy malnutrition is a consequence of inadequate nutrition, leading to developmental delays, increased susceptibility to infections, and childhood mortality (22). The prevalence of diet-related cancers, including stomach, colon, and breast cancers, highlights the impact of dietary patterns on neoplasms, whereas the high occurrence of hypertension underlines how dietary factors, such as salt and saturated fats, contribute to cardiovascular diseases (23). All these conditions, resulting from insufficient and imbalanced nutrition, significantly contribute to the loss of healthy life years and increase the overall burden of disease (24). These findings demonstrate that malnutrition influences not only individuals but also the health infrastructure of societies, underlining the necessity for countries to invest in health.

The widespread prevalence of nutrition-related health issues, coupled with insufficient investment in public health, prevents effective treatment of these conditions (25). This, in turn, shortens life expectancy at birth and reduces the number of healthy productive individuals in the population (26). Furthermore, insufficient healthcare spending per capita exacerbates the prevention and management of nutrition-related diseases, thereby worsening the general health status of the population and contributing to the increasing disease burden (27). Countries with a high disease burden and limited healthcare services are particularly vulnerable to health crises. Therefore, public health interventions and effective policies must be developed to address nutrition-related health issues. Priority areas for public health interventions and international support should be identified (28).

To better understand countries’ health and nutrition profiles this study focuses on the following three key themes: dietary habits, healthcare services and investments, and life expectancy and health outcomes. These themes enable us to assess countries’ nutritional and health status and use a clustering analysis approach to identify those at the highest risk. This study aimed to determine the most vulnerable countries based on the 2019 data from 168 countries and pinpoint priority areas for public health interventions.

## 2 Materials and methods

This study was designed as descriptive research to identify countries at risk based on indicators related to food consumption, diet-related diseases, healthcare services, and life expectancy. This study employed a cross-sectional analysis of secondary data.

### 2.1 Data and variables

The data used in this study were obtained from the Our World in Data platform (29–43). During data selection, emphasis was placed on using the most recent time frame, the largest number of countries, and variables relevant to the study’s focus. As a safeguard against sampling bias and spurious structure, the initial universe of 197 countries was screened for data completeness across all 24 study variables. Only the 168 countries with fully observed records were retained, thereby ensuring uniform information content within every cluster dimension. Cases exhibiting any missing value were removed rather than imputed, because our objective was to base the partitioning exclusively on verifiable empirical observations. Given the heterogeneity of the indicators—each derived from independent national statistics and global repositories—statistical imputation risked injecting artefactual covariance and distorting the true similarity patterns among countries. The most recent and comprehensive data from 2019 were used for the selected countries. The 24 variables relevant to this study were thematically categorized into the following three main groups:

Nutrition and Diet (NAD) group: this group comprises 14 variables representing individuals’ dietary habits and nutrition patterns. These variables include: animal protein (g/day), fruit (kg/year), vegetables (kg/year), milk excluding butter (kg/year), total fat (g/day), vegetal protein (g/day), total meat protein (g/day), egg protein (g/day), meat (kg/year), total protein (g/day), animal caloric intake (kcal/person), vegetal caloric intake (kcal/person), fat caloric intake (kcal/person), and carbohydrate share in daily caloric intake (%).

Health and Disease (HAD) group: this group focuses on HAD-related metrics and contains eight variables, including BMI-related deaths share, Alcohol-related deaths, Protein Energy Malnutrition Deaths rate, Anemia prevalence in pregnant women, Neoplasm cases per 100 people, Hypertension prevalence aged 30–79, CVD cancer diabetes CRD mortality age 30–70, and DALYs All causes rate.

Healthcare and Life Expectancy (HALE) group: this group comprises three core variables related to general healthcare services and life expectancy, including UHC Service Coverage Index, Life expectancy at birth, and Health expenditure per capita PPP.

The NAD group includes variables directly impacting individuals’ overall health based on food consumption. These variables reflect not only the quantity and types of foods consumed daily but also the quality of dietary habits and nutritional diversity. The HAD group encompasses metrics that capture the effects of nutrition and dietary patterns on health, particularly regarding chronic diseases. Lastly, the HALE group represents the quality of general healthcare services through indicators, such as healthcare expenditures and coverage, while reflecting the impact on life expectancy.

### 2.2 Data analysis

A scaling process was applied because the variables in the dataset had different variances and mean values. Each variable was standardized by subtracting its mean value.

The Elbow method was employed to identify the ideal number of clusters for the analysis. This method calculates the total within-cluster sum of squares (WSS) to determine the optimal k value in the k-means algorithm by measuring the distance of each data point from its cluster center. A graph of total within-cluster variance was generated for different k values, and the “elbow point” was identified, where the reduction in variance begins to plateau, indicating the appropriate number of clusters (44).


$$\text{Elbow Method}:W(k)=\sum_{i=1}^{k}\sum_{x\in C_{i}}\|x-\mu_{i}\|2$$

After determining the number of clusters, the k-means algorithm was independently applied to each variable group, following a machine learning-based distribution approach (45).


$$\begin{array}{c} \text{argmi}n_{j\in \{1,2,3\}}\|x_{i}-c_{j}\|2\\ c_{j}=\frac{1}{|S_{j}|}\sum_{x_{i}\in S_{j}}x_{i} \end{array}$$

In this process, the multiview clustering approach was applied separately to each group, with the similarity between the resulting clusters measured using the Jaccard similarity coefficient (46).


$$\mathrm{Sij}=\frac{\sum_{k=1}^{n}x_{\mathrm{ijk}}}{\sum_{k=1}^{n}y_{\mathrm{ijk}}}\to J(x\cap y)\frac{x\cap y}{x\cup y}$$

Cluster-number selection: The optimal number of clusters was determined primarily through the Elbow criterion, which provided a clear inflection point at k = 3 across all three thematic domains. To further validate cluster cohesion and separation, silhouette coefficients were computed for cluster counts ranging from k = 2 to k = 6. Among all configurations, the highest silhouette score (0.549) was observed for k = 3 in the Healthcare and Life Expectancy (HALE) group, supporting the robustness of this clustering structure. While the Nutrition and Diet (NAD) and Health and Disease (HAD) groups showed slightly higher scores for alternative k values (e.g., k = 2 or 4), the silhouette values for k = 3 remained comparable, falling within an acceptable range (0.284 and 0.322, respectively). Overall, silhouette coefficients across all models ranged from 0.233 to 0.549, indicating moderate-to-strong cluster quality. Given the statistical adequacy of the three-cluster solution and its interpretative alignment with our study design, we retained k = 3 for all analyses. Additionally, thematic concordance across the three independent k-means runs was assessed using the Jaccard similarity coefficient, which informed the late-integration step to identify countries consistently appearing in high-risk clusters across all domains.

The clusters with the highest similarity (Cluster 3) represented the countries with the lowest nutrition and health outcomes. Intersection analysis was performed for these clusters, and those with significant similarities were merged through a late integration process. A Venn diagram was used to visualize the intersections of the clusters. Normality was evaluated using the Shapiro–Wilk test to identify the variables contributing to the separation of clusters. As normality was not assumed for all variables (*p* < 0.05), robust regression using Maronna and Yohai’s MM estimator was employed for calculating the R^2^ value. The Kruskal–Wallis H test was employed to determine statistical significance. The analysis was conducted using RStudio (version 2023.06.2 + 561; Posit, PBC, Boston, MA, USA), incorporating several packages, including tidyverse, cluster, ggplot2, proxy, plotly, MASS, and ggvenn, to facilitate data processing and visualization (47–52).

## 3 Results

**Figure 1:**
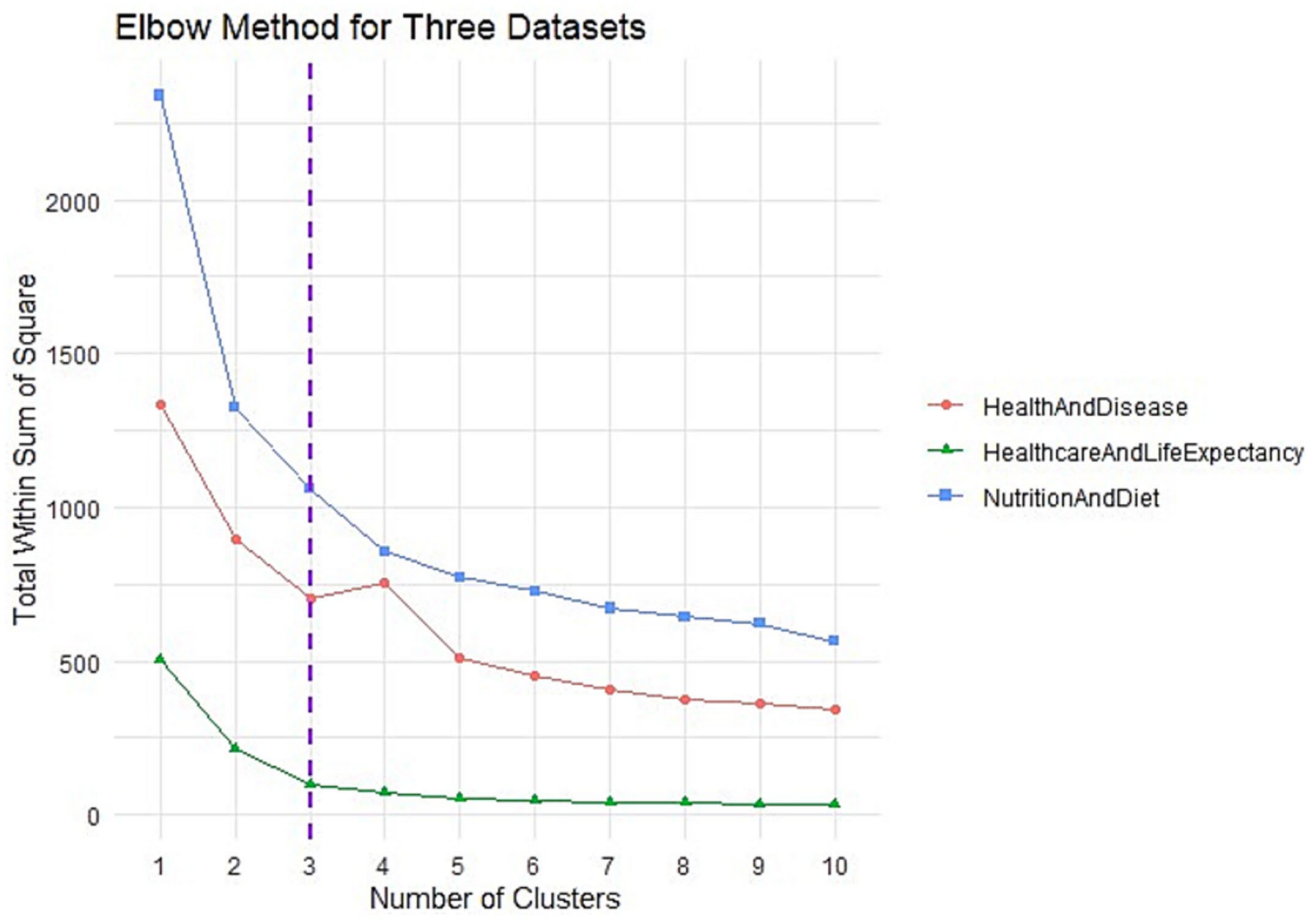
Elbow results.

The results of the Elbow method used for determining the optimal number of clusters for the three different groups revealed that the inflection point occurred at the second cluster for each group (Figure 1). This inflection point indicates where the total WSS begins to flatten, signifying the optimal clustering solution. A clear leveling-off or plateau was observed for all of the three groups after the third point. This transition is highlighted with dashed purple lines in Figure 1. These results suggest that the optimal number of clusters for all three groups is 3 and that the clustering solution becomes more stable at this point.

**Figure 2:**
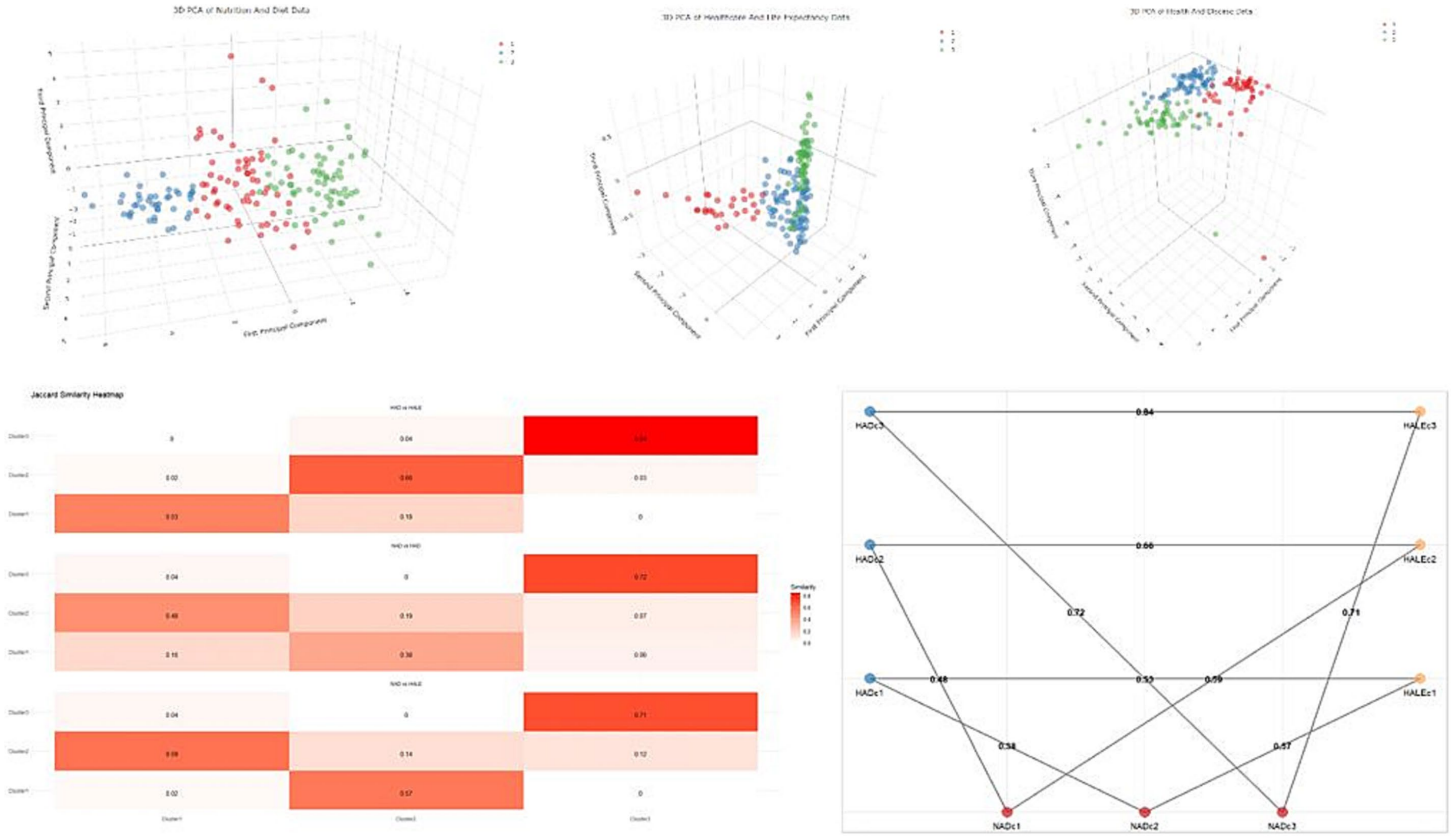
Distributions of K-means analyses and similarity of clusters in different groups. NAD, Nutrition and Diet; HAD, Health and Disease; HALE, Health and Life Expectancy; c, cluster.

A multiclustering analysis with three clusters for each group was subsequently performed using the k-means method. The results were processed through machine learning techniques and reduced to three dimensions using principal component analysis (Figure 2). After determining the cluster distributions, the Jaccard similarity coefficient was applied to evaluate the similarities between clusters across different groups. Cluster 3 in each group exhibited the highest degree of similarity, and these findings are illustrated in the heatmap in Figure 2. Specifically, the similarity between Cluster 3 of the NAD and HAD groups was 0.72, between the NAD and HALE groups was 0.71, and between HAD and HALE was 0.84. Similarity scores for other clusters ranged between 0 and 0.66 (Figure 2). These analyses demonstrate that Cluster 3 represents the highest-risk countries across the three groups, with a high degree of similarity.

Table 1 presents the distribution of countries across clusters resulting from the k-means analyses. In the NAD group, Clusters 1, 2, and 3 comprised 61, 42, and 65 countries, respectively. In the HAD group, Clusters 1, 2, and 3 comprised 45, 69, and 54 countries, respectively. In the HALE group, Clusters 1, 2, and 3 encompassed 27, 88, and 53 countries, respectively.

**Table 1:** Distribution of countries across clusters by group.

Nutrition and Diet group clustering analysis		
Cluster 1 (*N* = 61)	Cluster 2 (*N* = 42)	Cluster 3 (*N* = 65)
Algeria, Antigua and Barbuda, Armenia, Azerbaijan, Bahamas, Barbados, Belize, Bolivia, Bosnia and Herzegovina, Bulgaria, Chile, China, Colombia, Costa Rica, Cuba, Dominican Republic, Ecuador, El Salvador, Fiji, Gabon, Georgia, Grenada, Guyana, Iran, Jamaica, Japan, Kiribati, Kuwait, Kyrgyzstan, Lebanon, Malaysia, Maldives, Malta, Mauritius, Mexico, Moldova, Morocco, Myanmar, North Macedonia, Oman, Panama, Papua New Guinea, Paraguay, Peru, Qatar, Saint Lucia, Saint Vincent and the Grenadines, Samoa, Saudi Arabia, Serbia, Seychelles, South Africa, Suriname, Tajikistan, Trinidad and Tobago, Tunisia, Turkmenistan, Ukraine, United Arab Emirates, Uzbekistan, and Vietnam	Argentina, Australia, Austria, Bahrain, Belarus, Belgium, Brazil, Canada, Croatia, Cyprus, Czechia, Denmark, Estonia, Finland, France, Germany, Greece, Hungary, Iceland, Ireland, Israel, Italy, Kazakhstan, Latvia, Lithuania, Luxembourg, Mongolia, Montenegro, Netherlands, New Zealand, Norway, Portugal, Romania, Russia, Slovakia, Slovenia, Spain, Sweden, Switzerland, United Kingdom, the United States, and Uruguay	Afghanistan, Angola, Bangladesh, Benin, Bhutan, Botswana, Burkina Faso, Burundi, Cambodia, Cameroon, Cape Verde, Central African Republic, Chad, Comoros, Congo, Cote d’Ivoire, Democratic Republic of Congo, Djibouti, East Timor, Egypt, Eswatini, Ethiopia, Gambia, Ghana, Guatemala, Guinea, Guinea-Bissau, Haiti, Honduras, India, Indonesia, Iraq, Jordan, Kenya, Laos, Lesotho, Liberia, Madagascar, Malawi, Mali, Mauritania, Mozambique, Namibia, Nepal, Nicaragua, Niger, Nigeria, Pakistan, the Philippines, Rwanda, Sao Tome and Principe, Senegal, Sierra Leone, Solomon Islands, South Sudan, Sri Lanka, Sudan, Tanzania, Thailand, Togo, Uganda, Vanuatu, Venezuela, Zambia, and Zimbabwe
Health and Disease group clustering analysis		
Cluster 1 (N = 45)	Cluster 2 (N = 69)	Cluster 3 (N = 54)
Australia, Austria, Belgium, Bolivia, Canada, Chile, China, Colombia, Costa Rica, Cyprus, Denmark, Ecuador, El Salvador, Finland, France, Germany, Greece, Guatemala, Honduras, Iceland, Iran, Ireland, Israel, Italy, Japan, Luxembourg, Maldives, Malta, Mexico, Netherlands, New Zealand, Nicaragua, Norway, Panama, Peru, the Philippines, Portugal, Spain, Sri Lanka, Sweden, Switzerland, Thailand, United Kingdom, the United States, and Vietnam	Afghanistan, Algeria, Antigua and Barbuda, Argentina, Armenia, Azerbaijan, Bahamas, Bahrain, Barbados, Belarus, Belize, Bosnia and Herzegovina, Botswana, Brazil, Bulgaria, Cape Verde, Croatia, Cuba, Czechia, Dominican Republic, Egypt, Estonia, Fiji, Georgia, Grenada, Guyana, Hungary, Iraq, Jamaica, Jordan, Kazakhstan, Kuwait, Kyrgyzstan, Latvia, Lebanon, Lithuania, Malaysia, Mauritius, Moldova, Mongolia, Montenegro, Morocco, Namibia, North Macedonia, Oman, Paraguay, Qatar, Romania, Russia, Saint Lucia, Saint Vincent and the Grenadines, Samoa, Saudi Arabia, Serbia, Seychelles, Slovakia, Slovenia, South Africa, Sudan, Suriname, Tajikistan, Trinidad and Tobago, Tunisia, Turkmenistan, Ukraine, United Arab Emirates, Uruguay, Uzbekistan, and Venezuela	Angola, Bangladesh, Benin, Bhutan, Burkina Faso, Burundi, Cambodia, Cameroon, Central African Republic, Chad, Comoros, Congo, Cote d’Ivoire, Democratic Republic of Congo, Djibouti, East Timor, Eswatini, Ethiopia, Gabon, Gambia, Ghana, Guinea, Guinea-Bissau, Haiti, India, Indonesia, Kenya, Kiribati, Laos, Lesotho, Liberia, Madagascar, Malawi, Mali, Mauritania, Mozambique, Myanmar, Nepal, Niger, Nigeria, Pakistan, Papua New Guinea, Rwanda, Sao Tome and Principe, Senegal, Sierra Leone, Solomon Islands, South Sudan, Tanzania, Togo, Uganda, Vanuatu, Zambia, and Zimbabwe
Healthcare and Life Expectancy group clustering analysis		
Cluster 1 (N = 27)	Cluster 2 (N = 88)	Cluster 3 (N = 53)
Australia, Austria, Belgium, Canada, Cyprus, Czechia, Denmark, Finland, France, Germany, Iceland, Ireland, Israel, Italy, Japan, Luxembourg, Malta, Netherlands, New Zealand, Norway, Portugal, Slovenia, Spain, Sweden, Switzerland, United Kingdom, and the United States	Algeria, Antigua and Barbuda, Argentina, Armenia, Azerbaijan, Bahamas, Bahrain, Barbados, Belarus, Belize, Bhutan, Bolivia, Bosnia and Herzegovina, Brazil, Bulgaria, Cambodia, Cape Verde, Chile, China, Colombia, Costa Rica, Croatia, Cuba, Dominican Republic, Ecuador, Egypt, El Salvador, Estonia, Fiji, Georgia, Greece, Grenada, Guatemala, Guyana, Honduras, Hungary, India, Indonesia, Iran, Iraq, Jamaica, Jordan, Kazakhstan, Kuwait, Kyrgyzstan, Latvia, Lebanon, Lithuania, Malaysia, Maldives, Mauritius, Mexico, Moldova, Mongolia, Montenegro, Morocco, Nicaragua, North Macedonia, Oman, Panama, Paraguay, Peru, the Philippines, Qatar, Romania, Russia, Saint Lucia, Saint Vincent and the Grenadines, Samoa, Sao Tome and Principe, Saudi Arabia, Serbia, Seychelles, Slovakia, South Africa, Sri Lanka, Suriname, Tajikistan, Thailand, Trinidad and Tobago, Tunisia, Turkmenistan, Ukraine, United Arab Emirates, Uruguay, Uzbekistan, Venezuela, and Vietnam	Afghanistan, Angola, Bangladesh, Benin, Botswana, Burkina Faso, Burundi, Cameroon, Central African Republic, Chad, Comoros, Congo, Cote d’Ivoire, Democratic Republic of Congo, Djibouti, East Timor, Eswatini, Ethiopia, Gabon, Gambia, Ghana, Guinea, Guinea-Bissau, Haiti, Kenya, Kiribati, Laos, Lesotho, Liberia, Madagascar, Malawi, Mali, Mauritania, Mozambique, Myanmar, Namibia, Nepal, Niger, Nigeria, Pakistan, Papua New Guinea, Rwanda, Senegal, Sierra Leone, Solomon Islands, South Sudan, Sudan, Tanzania, Togo, Uganda, Vanuatu, Zambia, and Zimbabwe

In the NAD group, Cluster 1 exhibited average values of 40.21 g/day, 271.24 kg/year, and 307.03 kg/year for animal protein, fruit, and vegetable consumption, respectively. Cluster 2, with 64.08 g/day of animal protein consumption and 186.70 kg/year of milk consumption, indicated a higher intake of animal products. In contrast, Cluster 3 showed the lowest dietary intake, with 16.72 g/day of animal protein and other lower values (Table 2). These clusters revealed significant differences in food consumption among countries, with Cluster 3 showing the lowest nutrition profile.

**Table 2:** Average values for key indicators by cluster.

Clusters	Nutrition and diet			Health and disease			Healthcare and life expectancy		
Indicators	C1 (x̄)	C2 (x̄)	C3 (x̄)	C1 (x̄)	C2 (x̄)	C3 (x̄)	C1 (x̄)	C2 (x̄)	C3 (x̄)
Daily animal protein intake	40.21	64.08	16.72	53.27	42.26	16.99	65.91	40.58	16.60
Fruit consumption over a 2-year period (in kg)	271.24	222.76	159.73	231.89	239.28	172.94	227.92	243.50	164.19
Vegetable consumption over a 3-year period (in kg)	307.03	284.53	138.84	251.06	309.50	130.57	267.93	289.77	131.51
Annual milk consumption excluding butter (in kg)	70.70	186.70	30.58	127.86	103.31	23.33	177.03	90.24	26.80
Daily total fat intake	90.72	142.42	55.67	117.75	99.40	55.12	144.96	94.33	55.08
Daily vegetable protein intake	45.58	41.68	44.54	42.92	45.04	44.20	41.99	44.90	44.17
Daily total meat protein intake	19.88	28.29	7.07	23.59	20.23	7.46	28.19	19.22	7.70
Daily egg protein intake	2.59	3.73	0.70	3.49	2.52	0.54	3.93	2.64	0.42
Daily total protein intake	85.79	105.76	61.26	96.19	87.30	61.19	107.90	85.48	60.77
Per person caloric intake from animal sources	160.83	256.30	66.88	213.09	169.04	67.96	263.65	162.33	66.40
Per person caloric intake from vegetable sources	182.32	166.73	178.15	171.67	180.16	176.81	167.95	179.60	176.69
Per person caloric intake from fats	816.44	1281.76	501.03	1059.74	894.58	496.09	1304.60	848.96	495.68
Carbohydrate share in daily caloric intake	60.97	49.84	69.60	55.33	59.13	69.75	49.34	60.47	69.48
Annual meat consumption (in kg)	153.67	223.67	52.97	185.19	158.08	55.01	220.40	150.98	56.12
Alcohol-related deaths	14,106.96	17,352.66	6,073.58	26,720.38	6,968.97	12,448.01	16,848.10	14,103.25	5,739.49
Share of BMI-related deaths	14.56	11.64	8.14	9.87	15.83	6.84	9.36	14.43	7.23
Death rate due to protein malnutrition	2.40	0.50	10.82	1.84	1.55	12.51	0.41	2.04	12.68
Anemia prevalence in pregnant women	27.57	20.28	41.77	20.40	26.92	45.47	17.60	27.12	44.99
Cases of neoplasm per 100 individuals	7.45	9.47	4.72	7.38	8.81	4.05	8.73	7.80	4.47
Hypertension prevalence in individuals aged 30–79 years	39.19	35.98	36.69	30.74	42.79	36.19	31.12	39.97	36.54
Mortality rate due to cardiovascular diseases, cancer, diabetes, and chronic respiratory diseases in individuals aged 30–70 years	20.03	13.44	22.88	11.73	20.97	24.70	10.04	19.31	24.88
Disability-adjusted life years rate for all causes	29,853.20	22,348.54	46,629.87	22,321.79	30,299.22	50,012.61	19,323.07	28,942.38	51,340.81
Universal Health Coverage Index	70.07	79.98	49.71	79.59	69.17	46.48	84.48	70.24	45.33
Health expenditure per capita (PPP adjusted)	1263.02	4052.99	250.23	3568.45	1360.81	167.76	5422.25	1217.50	188.58
Life expectancy at birth	74.39	79.74	64.90	79.52	74.32	63.79	82.21	74.47	63.39

C, cluster.

In the HAD group, Cluster 1 was characterized by 213.09 g/day of animal-based calorie intake and a high alcohol-related death rate of %26.720.38. Cluster 2, with 20.23 g/day of meat protein intake and a %6.968.97 alcohol-related death rate, displayed slightly better health outcomes. Cluster 3, however, stood out owing to its low meat protein consumption of 7.46 g/day and a high disease burden, reflected by a disability-adjusted life year (DALY) rate of %50,012.61 (Table 2). These clusters showed that Cluster 3 in the HAD group had the lowest meat consumption and the highest disease burden.

In the HALE group, Cluster 1 stood out with a Universal Health Coverage (UHC) Service Coverage Index of 84.48 and a life expectancy of 82.21 years, indicating the highest access to healthcare services and quality of life among the clusters. Cluster 2, with a UHC index of 70.24 and a life expectancy of 74.47 years, represented moderate healthcare quality and access. In contrast, Cluster 3, with a UHC index of 45.33 and a life expectancy of 63.39 years, showed significantly lower access to healthcare services and life expectancy than the other clusters (Table 2). Therefore, Cluster 3 in the HALE group represented the lowest scores regarding healthcare access and life expectancy.

Table 3 summarizes the robust R^2^ values, Kruskal–Wallis χ^2^ statistics, and *p*-values for key variables in the NAD, HAD, and HALE groups are in. The robust R^2^ values indicate how much each variable contributes to the clustering solution, reflecting the proportion of variance explained within each group. The χ^2^ and p-values from the Kruskal–Wallis test show whether significant differences exist between clusters on the basis of these variables.

**Table 3:** Summary of explained variances for variables in the nutrition and diet, health and disease, and healthcare and life expectancy groups.

Nutrition and diet group				Health and disease group				Healthcare and life expectancy group			
Variables	Robust *R*^2^ value	χ^2^	*p*	Variables	Robust *R*^2^ value	χ^2^	*p*	Variables	Robust *R*^2^ value	χ^2^	*p*
Animal Protein (g/day)	0.831	142.12	1.38e-31*	Anemia Prevalence in Pregnant Women (%)	0.692	111.90	5.02e-25*	Health Expenditure per Capita (PPP)	0.793	129.20	8.81e-29*
Animal Caloric Intake (kcal/person)	0.831	142.12	1.38e-31*	Disability-Adjusted Life Years (DALYs) for All Causes (rate)	0.623	114.87	1.14e-25*	Life Expectancy at Birth (years)	0.796	132.76	1.49e-29*
Total Fat (g/day)	0.809	132.58	1.63e-29*	BMI-Related Deaths Share (%)	0.592	106.92	6.08e-24*	Universal Health Coverage (UHC) Service Coverage Index	0.834	134.33	6.76e-30*
Fat Caloric Intake (kcal/person)	0.809	132.58	1.63e-29*	Hypertension Prevalence (aged 30–79 years) (%)	0.572	99.71	2.23e-22*				
Meat Consumption (kg/year)	0.748	127.20	2.39e-28*	Mortality Rate for CVD, Cancer, Diabetes, and CRD (aged 30–70 years) (%)	0.437	85.28	3.03e-19*				
Total Meat Protein (g/day)	0.719	124.18	1.08e-27*	Protein–Energy Malnutrition Deaths Rate (%)	0.407	86.50	1.65e-19*				
Carbohydrate Share of Daily Caloric Intake (%)	0.705	119.16	1.33e-26*	Neoplasm Cases per 100 Individuals (rate)	0.369	84.54	4.39e-19*				
Total Protein (g/day)	0.694	119.53	1.11e-26*	Alcohol-Related Deaths (%)	0.033	15.61	4.09e-04*				
Milk (Excluding Butter) Consumption (kg/year)	0.597	92.03	1.04e-20*								
Egg Protein (g/day)	0.536	99.18	2.90e-22*								
Vegetable Consumption (kg/year)	0.162	50.64	1.01e-11*								
Fruit Consumption (kg/year)	0.112	32.25	9.94e-08*								
Vegetal Caloric Intake (kcal/person)	0.021	3.275	0.195								
Vegetal Protein (g/day)	0.021	3.275	0.195								

χ^2^: Kruskal–Wallis H test value; *: *p* < 0.001.

Among the variables in the NAD group, Daily Animal Protein Intake (g/day) and *Per Capita* Animal Caloric Intake (kcal/day) stood out, with R^2^ values of 0.831 and χ^2^ values of 142.12, making the strongest contributions to distinguishing clusters. Other notable variables included total fat intake (g/day) and *Per Capita* Fat Caloric Intake (kcal/day), both with robust R^2^ values of 0.809, indicating that fat intake is a key factor in differentiating nutritional profiles across countries. Additionally, Meat Consumption (kg/year) and Daily Meat Protein Intake (g/day), with R^2^ values of 0.748 and 0.719, respectively, played a significant role in the variation of nutritional profiles. All of these variables had highly significant *p*-values (*p* < 0.001), indicating meaningful differences between clusters. Conversely, Vegetal Caloric Intake (kcal/person) and Vegetal Protein (g/day) contributed only 2.1% to the clustering solution and did not produce significant effects (*p* > 0.05), suggesting that they are not strong factors in distinguishing between clusters.

In the HAD group, Anemia Prevalence in Pregnant Women (%) and DALY Rate for All Causes showed robust R^2^ values of 0.692 and 0.623, respectively, highlighting their significance in health-related clustering. Other significant variables included BMI-Related Death Rate (%) and Hypertension Prevalence (aged, 30–79 years) (%), with R^2^ values of 0.592 and 0.572, respectively, showing substantial effects in distinguishing clusters. The variable Alcohol-Related Deaths (%), although statistically significant (*p* < 0.001), had a relatively low R^2^ value (0.033), indicating a weak but still meaningful relationship with clustering.

In the HALE group, the UHC Service Coverage Index was the most critical variable, with a robust R^2^ value of 0.834 and a χ^2^ value of 134.33, playing a key role in healthcare-based clustering. Moreover, Life Expectancy at Birth (years) and *Per Capita* Health Expenditure (PPP) significantly contributed, with R^2^ values of 0.796 and 0.793, respectively, emphasizing the significance of healthcare access and financial investments in distinguishing between clusters (p < 0.001).

**Figure 3:**
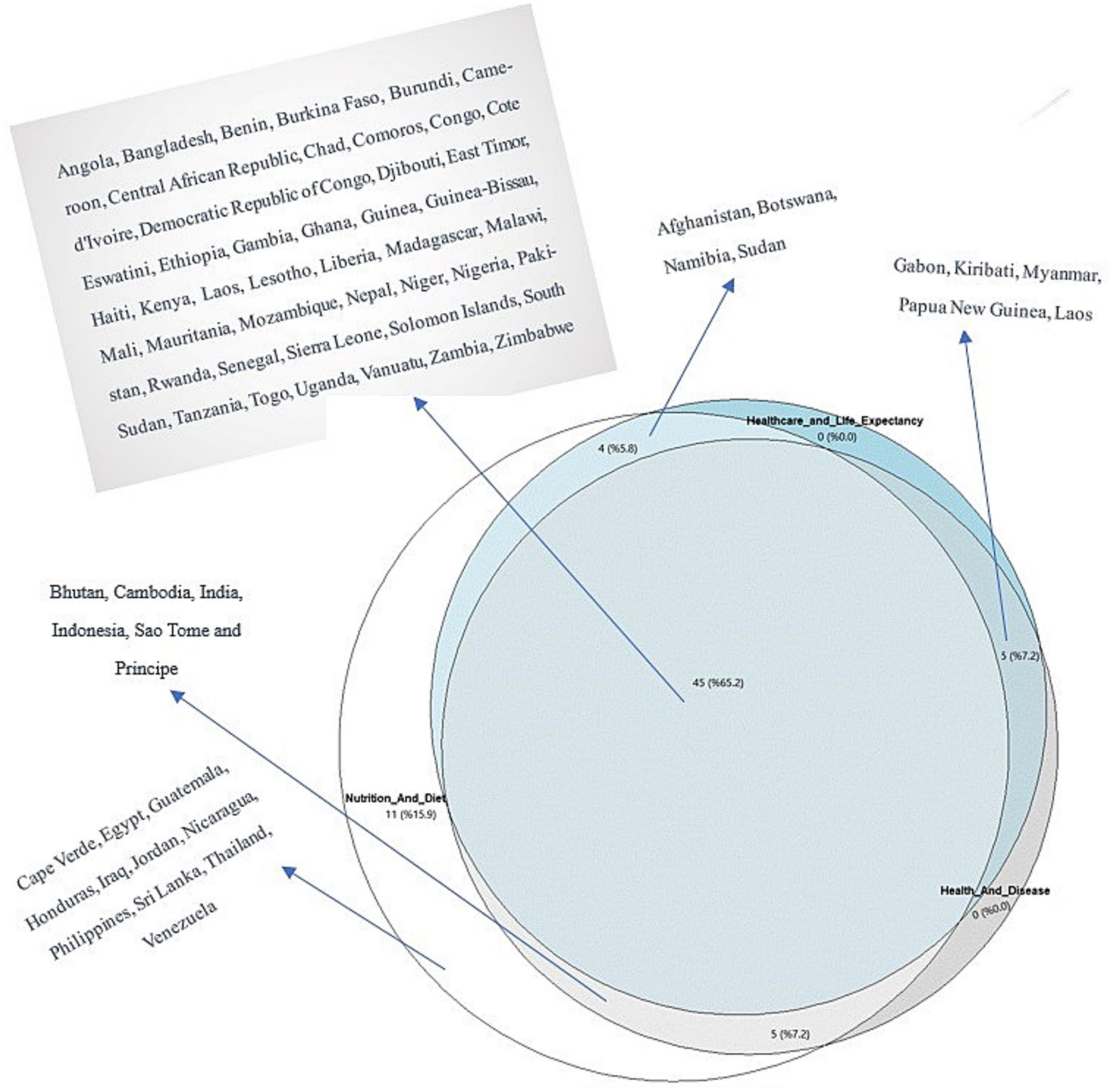
Late Integration combined with sets of countries.

The overlap and intersection of countries in Cluster 3 across the NAD, HAD, and HALE groups are illustrated in Figure 3. The union of these clusters comprised 69 countries (100.0%), whereas the intersection of all three groups consisted of 45 countries (65.2%). Countries in Cluster 3 of all three themes were identified as those at the highest risk across nutrition, health, and healthcare. These high-risk countries included Angola, Bangladesh, Benin, Burkina Faso, Burundi, Cameroon, Central African Republic, Chad, Comoros, Congo, Cote d’Ivoire, Democratic Republic of Congo, Djibouti, East Timor, Eswatini, Ethiopia, Gambia, Ghana, Guinea, Guinea-Bissau, Haiti, Kenya, Laos, Lesotho, Liberia, Madagascar, Malawi, Mali, Mauritania, Mozambique, Nepal, Niger, Nigeria, Pakistan, Rwanda, Senegal, Sierra Leone, Solomon Islands, South Sudan, Tanzania, Togo, Uganda, Vanuatu, Zambia, and Zimbabwe. These countries were categorized as the most vulnerable and were prioritized for global health interventions.

Outside the three-way intersection of the most vulnerable countries, other nations were identified within specific intersections or isolated groups. Eleven countries (15.9%) encountered health risks primarily due to specific nutritional and dietary habits or deficiencies. These countries were Cape Verde, Egypt, Guatemala, Honduras, Iraq, Jordan, Nicaragua, the Philippines, Sri Lanka, Thailand, and Venezuela. Additionally, five countries (7.2%)—Bhutan, Cambodia, India, Indonesia, and Sao Tome and Principe—were at considerable risks due to both nutritional deficiencies and the burden of health-related diseases. Four countries (5.8%)—Afghanistan, Botswana, Namibia, and Sudan—were at risk in terms of both NAD, as well as healthcare access and life expectancy. Finally, five countries (7.2%)—Gabon, Kiribati, Myanmar, Papua New Guinea, and Laos—were identified as being at risk both in terms of disease burden and limited healthcare access and life expectancy (Figure 3).

## 4 Discussion

This study reveals substantial global disparities in dietary habits, healthcare access, and life expectancy. The analysis shows that low-and middle-income countries, especially those in Africa and Asia (53), are at the highest risk for nutrition-related health challenges. The k-means clustering analysis identified countries such as Angola, Bangladesh, Nepal, Pakistan, and Burkina Faso as particularly vulnerable, due to their low protein intake, limited healthcare services, and short life expectancy. Nutrition-related diseases are prevalent in these countries, where access to healthcare is limited and food consumption profiles are poor. These results emphasize that improving health policies and identifying critical areas for public health interventions are urgently needed.

A key innovation of this study is its multi-dimensional approach, which simultaneously evaluates three major themes: dietary habits, healthcare access, and life expectancy. This comprehensive strategy for identifying health risks has not been widely applied in prior research and offers significant contributions to global public health policymaking.

Furthermore, our study thoroughly investigates the impact of low protein intake and malnutrition on chronic diseases. These findings align with prior research in the literature (9, 15). For example, Bahrami (54), emphasized that low protein intake accelerates cancer development in countries across Africa and Asia, where nutritional deficiencies are closely linked to high cancer rates. The prevalence of low protein consumption in these regions is exacerbated by food insecurity and low food safety, both of which are critical factors contributing to malnutrition. Food insecurity limits access to sufficient and diverse dietary protein sources, while low food safety increases the risk of foodborne illnesses, further impairing nutritional status (55, 56). These challenges create a vicious cycle of poor nutrition and health, disproportionately affecting vulnerable populations. Our findings also indicate that low protein consumption in these regions correlates with a higher prevalence of cancer. While Bahrami (54), focused solely on protein deficiencies, our study incorporates the additional dimension of limited healthcare access. This dual analysis allows us to assess the combined impact of nutritional and healthcare disparities on health outcomes.

Similarly, Prentice (57) highlighted the widespread prevalence of malnutrition and the high disease burden in low-income countries. Although this study solely focused on nutritional deficiencies, our study provides a more holistic analysis by examining both dietary habits and healthcare access. This comprehensive approach distinguishes our study from previous studies in literature, enabling a deeper understanding of the combined impact of malnutrition and healthcare access on life expectancy.

The disparities in food consumption, disease burden, and access to healthcare across countries worldwide are stark. Our analysis revealed that 37 of the highest-risk countries are in Africa. The Solomon Islands and Vanuatu are identified In Oceania, whereas Haiti stands out in the Caribbean. Countries such as Bangladesh, Laos, Nepal, Pakistan, and East Timor are at significant risk in Asia.

One study highlighted that in Pakistan, 19 million new cancer cases reported in 2020 were primarily attributed to the interaction between nutritional deficiencies and cancer development processes, underscoring that a thorough review of government policies is urgently needed (58). Another study observed that the new food security program in Haiti has had to address the country’s pressing food challenges, exacerbated by rising inflation and political instability, thereby triggering fuel shortages and severely disrupting the functioning of hospitals and other essential economic activities (59).

A study examining public financing for nutrition in Bhutan, Nepal, and Sri Lanka reported a downward trend in Nepal’s gross domestic product (GDP) from 2016 to 2018. The same study revealed that the budget allocation of Nepal for nutrition-specific public spending peaked at approximately 70% in 2017 but declined to 41% by 2018 (60).

A study conducted in 2022 revealed that out-of-pocket healthcare expenditures in Bangladesh exceeded the catastrophic threshold of 40%, with 10% of households forced to spend at this level, frequently on non-essential items (61). Additionally, the study highlighted that 8.61 million individuals (4.5% of the population) were living in poverty. Furthermore, a cross-sectional study in Bangladesh reported that in the absence of comprehensive social security programs supporting prenatal care for mothers, providing daycare for children, and addressing child nutrition, stunting prevalence among children reached approximately 19–20% (62).

Another study investigating the prices and nutritional content of 671 food and beverage products across 177 countries revealed that the affordability of nutritious diets for the poor was the lowest in Sub-Saharan Africa (5%), whereas animal-based foods were only a significant dietary component in high-income countries (over 70% affordability) (63). In contrast, globally, deaths attributed to high BMI exceed 4 million cases (64). Deaths due to cardiovascular diseases and cancer are closely associated with high BMI (65), illustrating the double-sided health impacts of global inequality (3).

The risks posed by food insecurity and inequality were discussed at the 2021 World Food Systems Summit, emphasizing how healthy diets remain unaffordable for much of the global population. These challenges were linked to environmental sustainability risk factors (66). Moreover, unpredictable events, including the Ukraine–Russia war, threaten not only African countries but also developing and middle-income nations by placing them at risk of food insecurity (67). In low-and middle-income countries, the need for more inclusive health insurance systems is underscored by the growing burden of chronic diseases and the slow increase in life expectancy (68).

Even in Europe, 16 of 24 countries have reported that at least 10% of urban populations encounter income-based food insecurity (69). Worldwide, over 150 million children with disabilities struggle to access healthcare owing to a lack of medical personnel and insufficient financial resources, a problem that poses an additional risk, particularly in Africa (70).

Food insecurity causes nutritional deficiencies and significantly increases the demand for healthcare services and healthcare expenditures. A study by Palakshappa et al. (71) noted that families experiencing food insecurity in the United States had healthcare expenses 20% higher than those of food-secure families, resulting in an annual difference of $2,456. The study emphasized that food insecurity increases healthcare costs for all family members in families with mixed insurance coverage, thereby adding a broader burden to the healthcare system. Similarly, a study by Clemens et al. (72) conducted in Canada reported that food insecurity among children increased the utilization of healthcare services and related healthcare costs. Children experiencing food insecurity require more frequent hospital visits, emergency department usage, and prescription medications than their food-secure peers.

These findings align with those presented in our study and highlight that the burden on healthcare systems is substantially increased in countries with high levels of food insecurity. Our k-means clustering analysis revealed that in low-income countries, particularly in Africa and Asia, populations experiencing food insecurity have restricted access to healthcare, which is directly related to higher disease burdens. In our study, countries in Cluster 3, including Angola, Bangladesh, Nepal, Pakistan, and Burkina Faso, were notable for low protein intake, inadequate healthcare services, and low life expectancy. The limited access to healthcare in these countries has resulted in higher rates of nutrition-related diseases, placing overwhelming strain on their healthcare systems. The effects of nutritional deficiencies on children, as also observed in the findings of Clemens et al. (72), are particularly evident in these countries. Owing to food insecurity, children in these regions experience increased healthcare needs and greater risks due to inadequate healthcare services. This finding reflects the broader relationship between food insecurity and poor health outcomes, further exacerbated by weak healthcare infrastructures.

GDP growth and foreign aid have positively influenced various indicators in Africa, including the quality of life index and health expenditures (73). A study examining the effects of nutrition and maternal mortality on the quality of life in Nigeria between 1990 and 2017 revealed that maternal mortality negatively impacted life expectancy. In contrast, the depth of the food deficit and the coverage of vitamin A supplementation positively affected life expectancy (74). When developed from a bidirectional perspective, incorporating health results associated with wasting, stunting, micronutrient deficiencies, morbidity, mortality, and those related to overweight and obesity, models used for measuring the economic cost of malnutrition could better explain these health outcomes (75).

Access to healthcare has significantly influenced life expectancy. A global analysis by Galvani-Townsend et al. (76) reported that countries with publicly funded healthcare services had an average life expectancy of approximately 10 years longer than those without such access. The study highlighted that access to healthcare improves individual health and may also mitigate health inequalities driven by social determinants. Similarly, Hao et al. (77), conducted a long-term study in China and demonstrated that expanding access to healthcare significantly increased life expectancy among older adults.

These findings align with the results of our study, particularly for countries in Cluster 3. In our research, limited access to healthcare was identified as a key issue in low-income countries in Africa and Asia, leading to lower life expectancy. Inadequate access to healthcare services was observed in countries such as Bangladesh, Nepal, Pakistan, and Burkina Faso, resulting in significantly lower life expectancy than those of other countries. Our k-means clustering analysis results suggest that expanding healthcare services in these countries could reduce the disease burden and improve life expectancy and quality of life.

The following four key components explain the global proliferation of social and sustainable balanced diets: innovation, food safety, power, equity, ethics, and the passage of time (78). Conversely, 100 countries with rigid national dietary guidelines are noted, several of which remain unstandardized at the national level, despite being linked to global health and sustainable development initiatives (79). A study analyzing the cost-effectiveness of external interventions and national measures for 129 countries during the 2019–2030 period showed that vitamin A supplementation and lipid-based nutritional supplements effectively reduced stunting. Vitamin A supplements and cash transfers prevented wasting, whereas intermittent preventive treatment in pregnancy, iron and folic acid supplements for non-pregnant women, and multiple micronutrient supplements for pregnant women helped reduce anemia prevalence (80).

Overall, our findings contribute to the evolving discourse on global health inequities by offering a multi-faceted approach that moves beyond single-variable assessments. By triangulating diet, disease burden, and healthcare access, our study provides actionable insights for global health governance, particularly in identifying intervention priorities. This integrated approach complements and extends prior work by situating nutritional challenges within broader health system and policy contexts.

In conclusion, our study provides an in-depth analysis of the adverse effects of nutritional deficiencies and limited access to healthcare on public health in low-and middle-income countries. The findings underscore that food insecurity and insufficient healthcare investments significantly contribute to the burden of chronic diseases in these regions. Strategies include promoting local agriculture, increasing access to low-cost protein sources, and expanding healthcare services, as previously recommended by Vissamsetti et al. (81), are consistent with the findings of our study. These results serve as critical guidance for policymakers aiming to improve public health policies and reduce health inequalities.

For these populations, supporting local agriculture, improving access to affordable protein sources, and expanding healthcare services can result in long-term improvements in health outcomes. Furthermore, governments should implement nutrition and health education programs to encourage healthy habits within communities, which could be instrumental in preventing future health crises.

Recently, global healthcare systems have been significantly strained owing to major crises, including the coronavirus disease 2019 (COVID-19) pandemic and the Ukraine–Russia war. These crises have had devastating effects, particularly in low-and middle-income countries, which are already vulnerable regarding food security and healthcare access. Sokan-Adeaga et al. (82), highlighted that the COVID-19 pandemic significantly disrupted global supply chains, making it difficult for millions of individuals to access essential food items. Similarly, Miralles et al. (83) emphasized that, during the pandemic, several countries could not deliver necessary healthcare services due to overburdened health systems and resource shortages. This finding underscores the critical significance of healthcare system resilience despite global crises.

Moreover, the Ukraine–Russia war has posed a severe threat to global food security, as these two countries collectively supply 30% of the world’s grain. The conflict has left several countries experiencing food insecurity, particularly those in Africa and the Middle East, which heavily depend on wheat imports. The war has led to supply shortages, inflationary pressures, and soaring energy prices, which have increased food costs and the risk of hunger in poorer nations (84).

These crises highlight the critical need to enhance the resilience of both healthcare and food systems. Khatri et al. (85), suggested that policymakers should reassess national health and food security strategies to better prepare for future crises. Key strategies that could mitigate the impacts of such crises include encouraging local food production and strengthening healthcare systems in low-income countries.

Our study indicates that countries in different regions, including Africa, Asia, and Oceania, encounter distinct risks related to dietary habits, access to healthcare, and life expectancy. Social, economic, and political differences across regions influence these risk factors. For example, Africa is confronted with high levels of malnutrition and difficulties in accessing healthcare. Adeyeye et al. (86), noted that inadequate infrastructure and political instability in several African countries severely limit access to healthcare services, exacerbating the prevalence of nutrition-related diseases. Fluctuations in food prices and dependence on foreign aid are key factors that increase the risk of food insecurity in these economically low-income countries.

In Asia, rapid population growth, environmental degradation, and economic inequalities significantly influence the risk factors. Rao et al. (87), emphasized that the rapid population increase in South Asian countries immensely pressured healthcare systems, thereby leading to significant inadequacies in the health services available to communities. Additionally, industrialization and environmental degradation have negatively impacted food production, whereas water access-related issues further aggravate food insecurity in the region.

The most prominent risk factors in Oceania are geographic isolation and climate change. Cao et al. (88), explained that various island nations in this region heavily depend on food supply chains, and food security and healthcare access are severely compromised by the environmental threats posed by climate change. Global warming and rising sea levels threaten agricultural land, whereas natural disasters limit these countries’ capacity for self-sustained food production.

These varying regional risk factors provide critical insights into how health policies should be developed. Policymakers should consider regional differences when designing strategies for improving food security and healthcare access, ensuring that these strategies are tailored to the unique challenges encountered by each region. These recommendations are vital for protecting and improving public health in low-and middle-income countries and should be considered in shaping global health policies.

### 4.1 Limitations and future research

This study had several limitations. First, the data used in this study were from 2019, and owing to the lack of more recent data, the effects of the COVID-19 pandemic could not be considered. The pandemic may have led to significant changes in dietary habits and access to healthcare, especially in low-and middle-income countries. Second, the data used in our analyses were largely obtained from secondary sources, and the accuracy of the results may have been affected by data gaps or differences in reporting standards. Third, this study tended to generalize across countries, whereas each country’s nutrition and health dynamics can vary according to regional and cultural differences. Lastly, the k-means clustering analysis was based on certain assumptions regarding data clusters, and the results could vary when different clustering techniques were applied.

Future research can address these limitations and build on the current findings. First, studies exploring more recent data and long-term trends are warranted to better understand the impact of major crises, including the COVID-19 pandemic, on dietary habits and access to healthcare. Additionally, the findings based on secondary data sources can be strengthened by testing them against larger and more diverse datasets. A deeper analysis of the health and nutrition dynamics of developed countries and other regions could increase the generalizability of the results.

Moreover, more complex multivariate relationships in such analyses may be investigated by employing different clustering techniques and machine learning algorithms. This investigation would enhance the accuracy of the findings and help uncover more intricate relationships between health systems and dietary patterns. In conclusion, future research utilizing broader datasets and advanced analytical methods for examining these findings will contribute to the more effective shaping of global health policies.

## 5 Conclusion

Hunger and malnutrition not only devastate the body but also threaten the very foundations of civilization.

The findings of this study are anticipated to play a critical role in shaping health and nutrition policies at both national and international levels. International organizations and nations are recommended to intensify their efforts to reduce global inequalities in nutrition and health, creating a healthier and more equitable world.

This study explores the relationship between dietary habits, access to healthcare, and life expectancy on a global scale, revealing significant inequalities between these three factors, particularly in low-and middle-income countries. The analyses show that several countries in Africa and Asia are in the highest risk group for nutrition-related health risks. Low protein intake, inadequate healthcare services, and low life expectancy emphasize the urgent need to reevaluate health policies in these countries.

Developing global health strategies and implementing sustainable nutrition policies are crucial to reducing health disparities in these high-risk countries. Our study demonstrates that investments in healthcare access and nutrition policies can positively affect public health. To further investigate and develop more effective strategies for policymakers, future research can build on these findings.

Therefore, this study provides valuable data for improving health and nutrition policies in low-and middle-income countries and represents a significant step in shaping global health policies.

## Data Availability

Data supporting the findings of this study are available from the corresponding author upon reasonable request. The raw data sources used in this study are cited in “Data and Variables” section.
